# Caprini risk assessment model combined with D-dimer to predict the occurrence of deep vein thrombosis and guide intervention after laparoscopic radical resection of colorectal cancer

**DOI:** 10.1186/s12957-023-03183-7

**Published:** 2023-09-21

**Authors:** Wuming Zhang, Ruizheng Sun, Xianqin Hu, Zhikang Chen, Chen Lai

**Affiliations:** 1 Department of General Surgery, Xiangya Hospital of Central South University, Changsha, Hunan Province China; 2 Hunan Key Laboratory of Precise Diagnosis and Treatment of Gastrointestinal Tumor, Xiangya Hospital of Central South University, Changsha, Hunan Province China; 3 International Joint Research Center of Minimally Invasive Endoscopic Technology Equipment and Standardization, Xiangya Hospital of Central South University, Changsha, Hunan Province China; 4 National Clinical Research Center for Geriatric Disorders, Xiangya Hospital, Central South University, Changsha, Hunan Province China

**Keywords:** Deep vein thrombosis (DVT), Caprini risk assessment model, D-dimer, Colorectal cancer

## Abstract

**Background:**

To explore the diagnostic value of Caprini risk assessment model (2005) combined with D-dimer for deep vein thrombosis, and to exclude patients with low incidence of thrombosis who might not need anticoagulation after surgery.

**Methods:**

A total of 171 colorectal cancer patients who underwent surgery from January 2022 to August 2022 were enrolled in this study. Caprini risk assessment model was used to evaluate patients the day before surgery, and full-length venous ultrasonography of lower extremity was used to assess whether patients had thrombosis one day before surgery and the sixth day after surgery. The value of D-dimer was measured by enzyme-linked immunosorbent assays on the first day after surgery, and clinical data of patients were collected during hospitalization.

**Results:**

A total of 171 patients were divided into IPC Group and IPC + LMWH Group according to whether low molecular weight heparin (LMWH) were used to prevent thrombus after surgery. Eventually, 17.6% (15/85) patients in IPC Group and 7% (6/86) patients in IPC + LMWH Group developed DVT. Through separate analysis of IPC Group, it is found that Caprini score and D-dimer were independent risk factors for DVT (Caprini OR 3.39 [95% CI 1.38–8.32]; *P* = 0.008, D-Dimer OR 6.142 [95% CI 1.209–31.187]; *P* = 0.029). The area under ROC curve of Caprini risk assessment model is 0.792 (95% CI 0.69–0.945, *P* < 0.01), the cut-off value is 9.5, and the area under ROC curve of D-dimer is 0.738 (95%CI 0.555–0.921, *P* < 0.01), the cut-off value is 0.835 μg/mL, and the area under the ROC curve was 0.865 (95% CI 0.754–0.976, *P* < 0.01) when both of them were combined. Based on decision curve analysis, it is found that Caprini risk assessment model combined with D-dimer can benefit patients more. All patients are divided into four groups. When Caprini score < 10 and D-dimer < 0.835 μg/mL, only 1.23% (1/81) of patients have thrombosis and LMWH has little significance. When Caprini score > 10 and D-dimer > 0.835 μg/mL, the incidence of DVT is 38.7% (12/31) and LMWH should be considered.

**Conclusions:**

The Caprini risk assessment model and D-dimer can provide more accurate risk stratification for patients after laparoscopic radical resection of colorectal cancer.

## Background

Colorectal cancer is one of the most common malignant tumors worldwide, accounting for the third highest incidence and mortality of malignant tumors [1]. Laparoscopic surgery is currently the primary treatment for colorectal cancer and can considerably improve the prognosis of patients. Deep vein thrombosis (DVT) is a common postoperative complication in patients with colorectal cancer and a great burden on medical resources worldwide [2]. Postoperative DVT of patients with CRC has been a nonnegligible problem. The incidence of DVT after colorectal surgery is as high as 40% without prevention, and the main causes include advanced age, malignant tumors, major surgery, and bedridden [3].

At present, the main standpoint is to reduce the occurrence of DVT by effective prevention. A Japanese study on postoperative venous thromboembolism in patients with abdominal tumors found that the incidence of venous thromboembolism after intermittent pneumatic compression (IPC) prevention was 19.4% and that of thrombosis after enoxaparin prevention was 1.2%. Therefore, accurate evaluation and prompt interventions are important for reducing DVT [4].

The Caprini risk assessment model (2005) has been verified and is currently the most widely used model in surgery [5]. The American College of Chest Physicians guidelines recommend that abdominal surgery should be stratified through the Caprini risk assessment model and that mechanical prophylaxis should be combined with drug prophylaxis for high-risk patients (Caprini score ≥ 5) [6]. All patients with colorectal cancer who are stratified according to the Caprini risk assessment model are supposed to be at high risk postoperatively. The Caprini risk model might not solely be an accurate indicator for DVT occurrence and intervention in colorectal cancer.

D-dimer is a biomarker of fibrin formation and degradation and acts a marker of coagulation and fibrinolysis system activation. As an indirect marker of thrombosis activity, D-dimer is of great significance for checking the formation of acute venous thrombosis [7]. Although D-dimer has high negative predictive value for DVT, it has low positive predictive value and low specificity for thrombosis [8, 9]. We hope to re-stratify patients with colorectal cancer through the Caprini risk assessment model and postoperative D-dimer levels, thereby identifying people at high risk of thrombosis from patients with colorectal cancer and further guiding postoperative thrombus prevention strategies for these patients.

## Materials and methods

### Patients and design

Prospectively collect patients’ clinical data generated undergoing laparoscopic radical resection of colorectal cancer in department of Colorectal and Anal Surgery, Xiangya Hospital, Central South University from January 2022 to August 2022. The patients will be jointly assessed by two professionally trained doctors using Caprini risk assessment model one day before the surgery. The aim of study was to assess whether the patients had venous thrombosis by color Doppler ultrasonography of both lower extremity veins on day 6 (± 1) after surgery. This study was retrospective and did not intervene in the treatment process, approved by the Ethics Committee of Xiangya Hospital, Central South University Hospital (No. 202112186).

The inclusion criteria include.

1. Patients with informed consent.
2. Postoperative pathology examination confirmed colorectal cancer.
3. Patients undergoing elective laparoscopic radical resection of colorectal cancer.

The exclusion criteria include.

1. Patients who require the informed consent to be returned.
2. Patients who did not have color Doppler ultrasonography.

### Data collection

The basic information about patients was collected through the case system, such as sex and age. The following clinical information was collected: operation time, intraoperative blood loss, hospitalization days, body mass index, preoperative chemotherapy, preoperative intestinal obstruction, white blood cell count on admission, hemoglobin on admission, D-dimer on the first day postoperatively, color Doppler ultrasonic of veins of both lower extremities preoperatively, color Doppler ultrasonic of veins of both lower extremities on the sixth day postoperatively, results of the pathological examination, use of anticoagulants, and Caprini score. The cut-off of D-dimer was calculated based on ROC curves of the IPC group. The Youden index reached max when the cutoff of D-dimer was 0.835 μg/mL.

### Data analysis

SPSS (Version 26), GraphPad Prism (Version 8.0), and R (Version 4.0) were used for retrospective analysis. Analytical method for describing data and differential analysis depended on the data type. First, the normality test was conducted, the measurement data of normality were tested using independent sample T-test, the non-normal measurement data were tested using the Mann–Whitney U test, and the data were expressed by *x* ± *s* or M (P25–P75). The counting data were measured by the chi-square test and Fisher's exact test and expressed as percentages. Differential factors between patients with or without DVT in the IPC group were considered significant factors for DVT. *P* < 0.05 were considered significant. The power of statistic in this study was not calculated because the effect size was not previously confirmed. These significant factors were then fitted in the multivariate regression model to evaluate independence. Simultaneously, the receiver operating characteristic curves (ROC) were obtained to evaluate the applicability of the Caprini risk assessment model and D-dimer. The potential net benefit of different intervention thresholds based on predictive models was evaluated using the decision curve analysis (DCA). Construction and visualization of DCA were achieved in the R statistics environment with the guide of MSKCC (https://www.mskcc.org/departments/epidemiology-biostatistics/biostatistics/decision-curve-analysis).

## Results

### Basic data

**Fig. 1:**
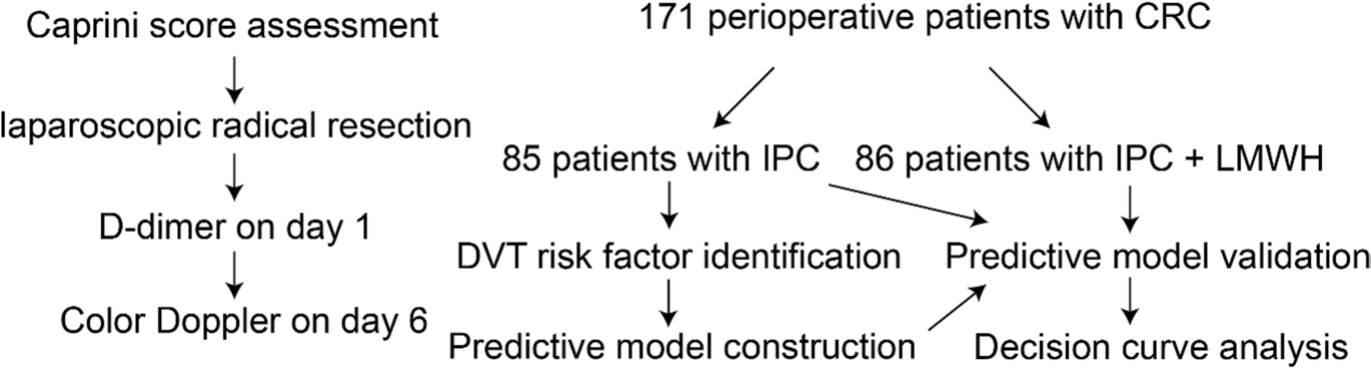
Schematic diagram of the study

The clinical data of 186 patients were collected, of which 171 patients were included and 15 patients excluded. The reasons for exclusion were as follows: 6 patients had a color Doppler ultrasound of lower extremity venous showing thrombosis preoperatively and nine patients had no preoperative or postoperative color Doppler ultrasound of lower extremity venous. Furthermore, 85 (49.4%) patients did not have a prophylactic use of anticoagulants, and 86 (50.6%) patients used of low molecular weight heparin (LMWH) to prevent thrombosis from the first day postoperatively (nadroparin 4100 IU, once a day) till discharge. If there was massive hemorrhage and the platelet count was < 60 × 10^12^, the anticoagulants were stopped. All patients were treated with IPC (twice a day, 20 min each time) to improve venous reflux and prevent venous thrombosis of the lower extremities (Table 1). In this way, patients were stratified into two groups, the IPC group and the IPC + LMWH group (Fig. 1).

**Table 1:** Patient baseline characteristics table

Subjects		IPC group	IPC + LMWH group	*P* value
Age		58.88(± 8.93)	60.93(± 10.07)	0.162
Sex	Man	48	54	0.4
	Woman	37	32	
Pre-operative chemotherapy	No	70	63	0.153
	Yes	15	23	
Incomplete intestinal obstruction	No	80	78	0.399
	Yes	5	8	
Pelvic operation	No	44	47	0.705
	Yes	41	39	
Postoperative hospital stays (day)		6 (5.5–7)	6 (6–8)	0.281
BMI		23.80 (21.3–25.7)	23.16 (21.0–25.0)	0.814
Caprini score		8.95(± 1.0)	9.45(± 1.37)	0.011
Operation time (min)		190 (152–240)	190 (168–230)	0.408
Intraoperative blood loss (mL)		50 (25–50)	50 (30–50)	0.606
Diameter of tumor (cm)		4 (2.5–5)	4 (3–5)	0.034
WBC (× 10^Λ9^/L)		5.1 (4.05–6.4)	4.9 (3.9–6)	0.203
HB (g/L)		125 (110.5–141)	120.5 (120.5–130)	0.076
PLA (× 10^Λ9^/L)		220 (173.5–268)	206.5 (164.5–259.75)	0.17
D-dimer (μg/mL)		0.51 (0.315–0.855)	0.755 (0.467–1.175)	0.003

In total, 21 patients developed DVT, including 17.6% (15/85) in the IPC group and 7% (6/86) in the IPC + LMWH group. The incidence of DVT in IPC + LMWH was significantly lower than IPC group (*P* = 0.038). All thrombosis occurred in the distal veins of the lower extremities but none in the proximal veins; all venous thromboses of the lower extremities were asymptomatic.

### Caprini risk assessment and D-dimer test were independent risk factors for DVT in the IPC group

To exclude the effect of LWMH, differential analyses were conducted between patients with or without DVT in the IPC group. The results revealed that a D-dimer level of > 0.835 μg/mL, Caprini score, tumor diameter, preoperative hemoglobin level, preoperative platelet count, and incomplete intestinal obstruction preoperatively were potentially risk factors for postoperative thrombosis (Table 2).

**Table 2:** Single-factor analysis of deep vein thrombosis incidence risk in IPC group

Thrombus occurrence			
Subjects	No	Yes	*P* value
Sex			
Male	41	7	0.399
Female	29	8	
Pre-operative chemotherapy			
No	56	14	0.289
Yes	14	1	
Incomplete intestinal obstruction			
No	68	12	0.037
Yes	2	3	
D-dimer (μg/mL)			
< 0.835	58	4	< 0.01
> 0.835	12	11	
Pelvic operation			
No	34	10	0.203
Yes	36	5	
Age	57 (52–60.5)	67 (61–80)	< 0.01
BMI	23.8 (23.6–25.5)	24.0 (19.5–29.5)	0.708
Postoperative hospital stays (day)	6 (5.75–7)	6 (5–9)	0.365
Caprini score	9 (8–9)	10 (10–11)	< 0.01
Operation time	195 (158–240)	180 (145–260)	0.782
Intraoperative blood loss	50 (27.5–50)	50 (20–100)	0.57
Diameter of tumor	3.55 (2.48–4.55)	4.5 (4–7)	0.014
WBC × 10^Λ9^/L	5.2 (4.1–6.45)	4.6 (3.5–5.5)	0.271
HB g/L	128 (114–142)	110 (81–127)	0.014
PLA × 10^Λ9^/L	215.5 (166–254)	240 (201–314)	0.08

The Caprini score was included and age was excluded in binary logistics regression due to the linear relationship between age and the Caprini score. Preoperative hemoglobin level, preoperative platelet count, tumor diameter, a D-dimer level of > 0.835 μg/mL, and obstruction were included in multivariate analysis. The results showed that D-dimer (*P* = 0.029, OR = 6.142, CI (1.209–31.187)) and Caprini score (*P* < 0.01, OR = 3.39, CI (1.38–8.32) were the independent influencing factors of postoperative thrombosis. The incidence of DVT with a D-dimer level of > 0.835 was 6.142 times higher than that of a D-dimer level of < 0.835 μg/mL. The incidence of DVT increased by 3.39 times when the Caprini score increased by one point (Table 3).

**Table 3:** Logistic regression analysis of deep vein thrombosis risk factors

Risk factor	*P*	OR	95%CI	
Caprini score	0.008	3.39	1.38	8.32
D-dimer	0.029	6.142	1.209	31.187
Preoperative platelet	0.450	1.004	0.994	1.014
Preoperative hemoglobin	0.273	0.983	0.953	1.014
Tumor diameter	0.321	1.256	0.801	1.970
Intestinal obstruction	0.227	0.207	0.016	2.660
Constant	0.057	0		

### Models based on Caprini risk assessment and D-dimer showed great discrimination of DVT in the IPC group

**Fig. 2:**
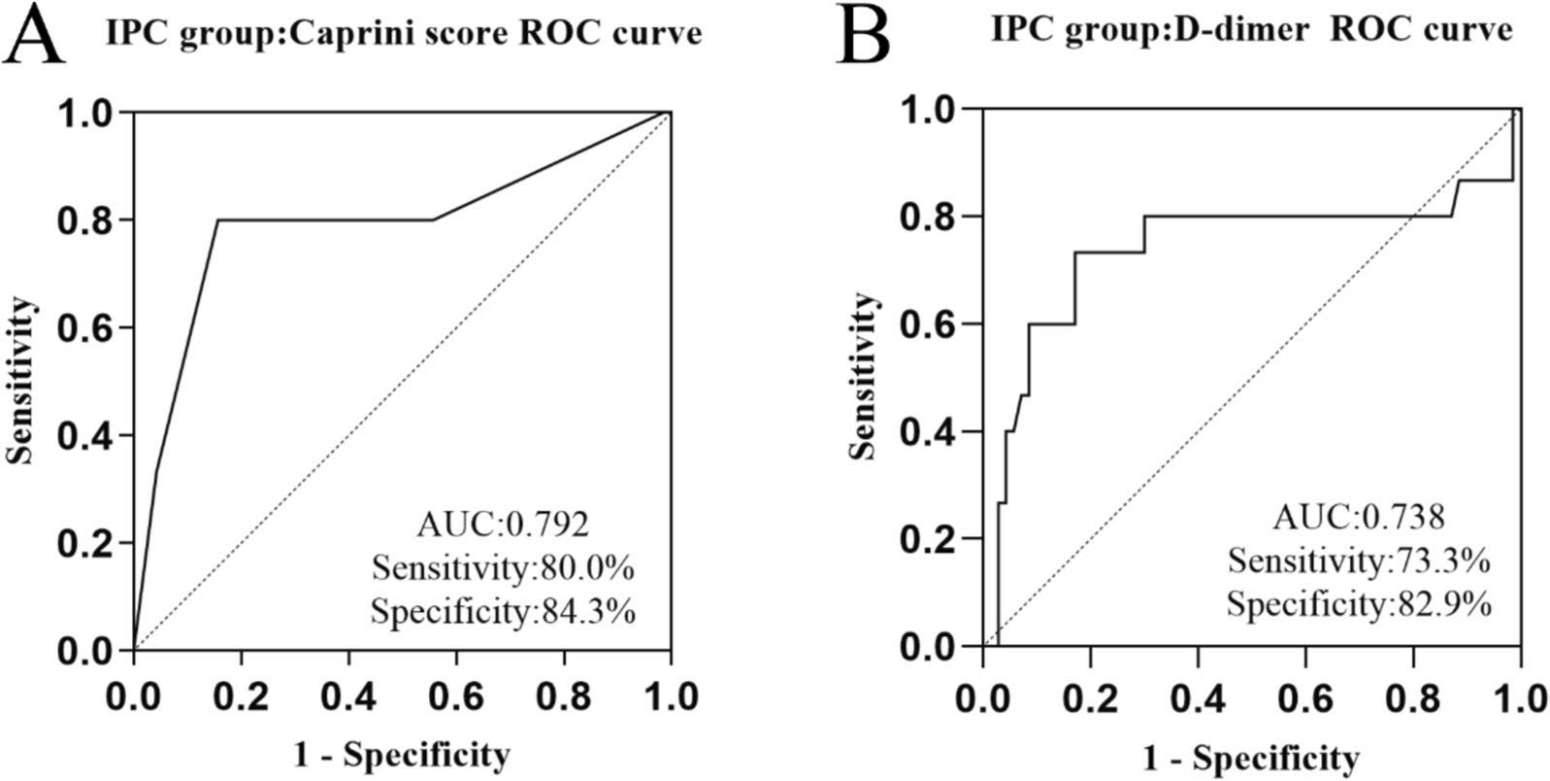
**A** In the IPC group, the area under Caprini risk assessment model is 0.792, and the optimal cut-off is 9.5. **B** In IPC group, the area under D-dimer ROC curve was 0.738, and the optimal cut-off is 0.835 μg/ml

In the IPC group, ROC based on Caprini risk assessment or D-dimer revealed considerable predictive ability of DVT. Area under curve (AUC) of the Caprini model was 0.792 (95% confidence interval (CI) 0.69–0.945, *P* < 0.01), the cutoff value was 9.5, the sensitivity was 80%, and the specificity was 84.3% (Fig. 2). The AUC of D-dimer was 0.738 (95% CI 0.555–0.921, *P* < 0.01), the cutoff value was 0.835 μg/mL, the sensitivity was 73.3%, and the specificity was 82.9% (Fig. 2).

**Fig. 3:**
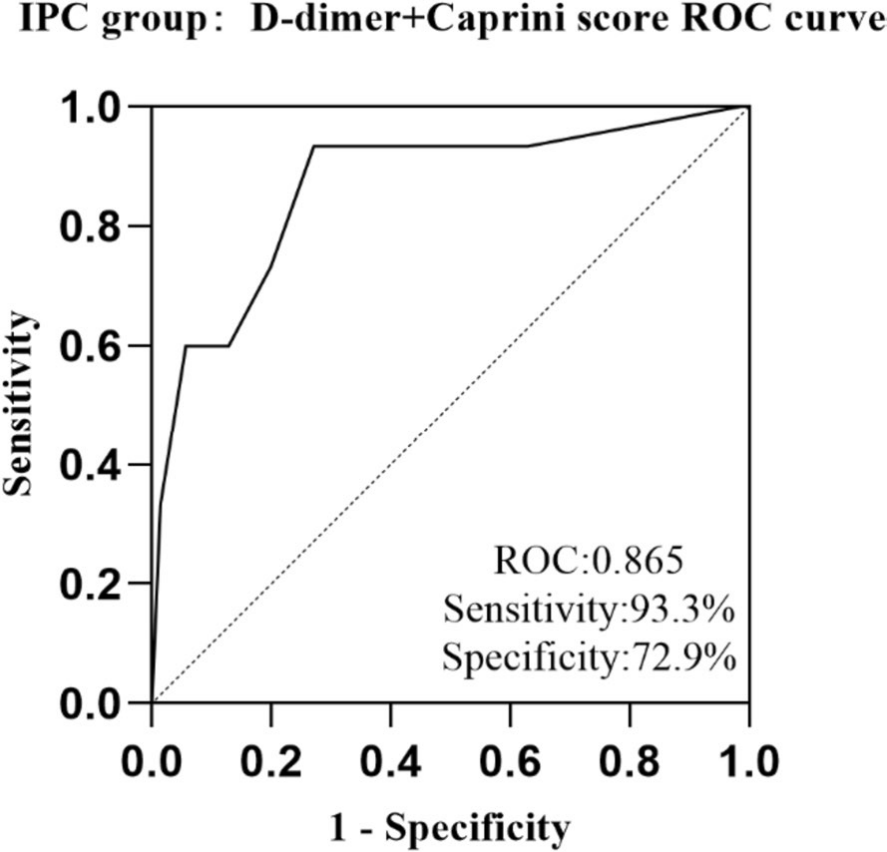
In the IPC group, D-dimer combined with Caprini risk assessment model was used to predict patients developed deep vein thrombosis. The area under ROC curve was 0.865

We then integrated Caprini score as a continuity variable and D-dimer as a binary variable into a novel predictive model according to the results of multivariate logistic regression model. A new ROC curve based on Caprini score and D-dimer was obtained. The AUC was 0.865 (95% CI 0.754–0.976), the sensitivity was 93.3%, and the specificity was 73.9% (Fig. 3). The Caprini score combined with the D-dimer level was a better predictor of postoperative DVT than the Caprini score or D-dimer alone in patients with colorectal cancer.

### The model based on Caprini score and D-dimer was reliable in the whole cohort

**Fig. 4:**
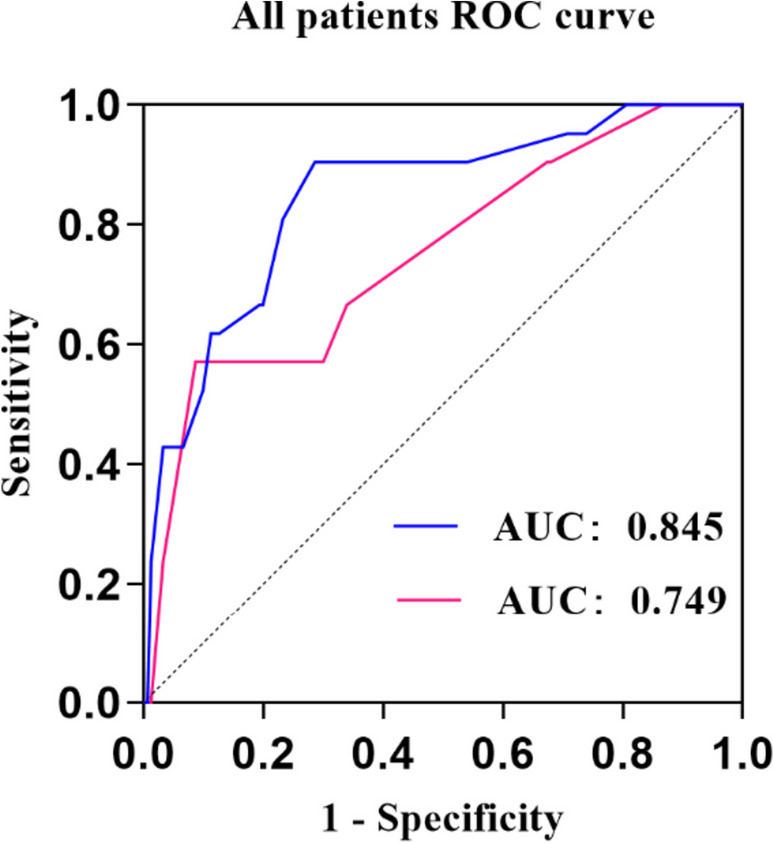
In all patients, the red curve represents Caprini risk assessment model and LMWH predict the occurrence of deep vein thrombosis. The blue curve represents Caprini risk assessment model and LMWH combined with D-dimer predict the occurrence of deep vein thrombosis

We then observed the predictive robustness of Caprini score and D-dimer. The Caprini score and anticoagulants were used to predict the occurrence of postoperative DVT in the whole population. The AUC was 0.750 (95% CI 0.632–0.868, *P* < 0.01). After adding D-dimer in the model, the AUC reached 0.845 (95% CI 0.754–0.936, *P* < 0.01), indicating that the Caprini score combined with the D-dimer still had promising predictive efficiency in the whole population (Fig. 4).

### Clinical decisions based on the model of Caprini score and D-dimer might benefit for patients

**Fig. 5:**
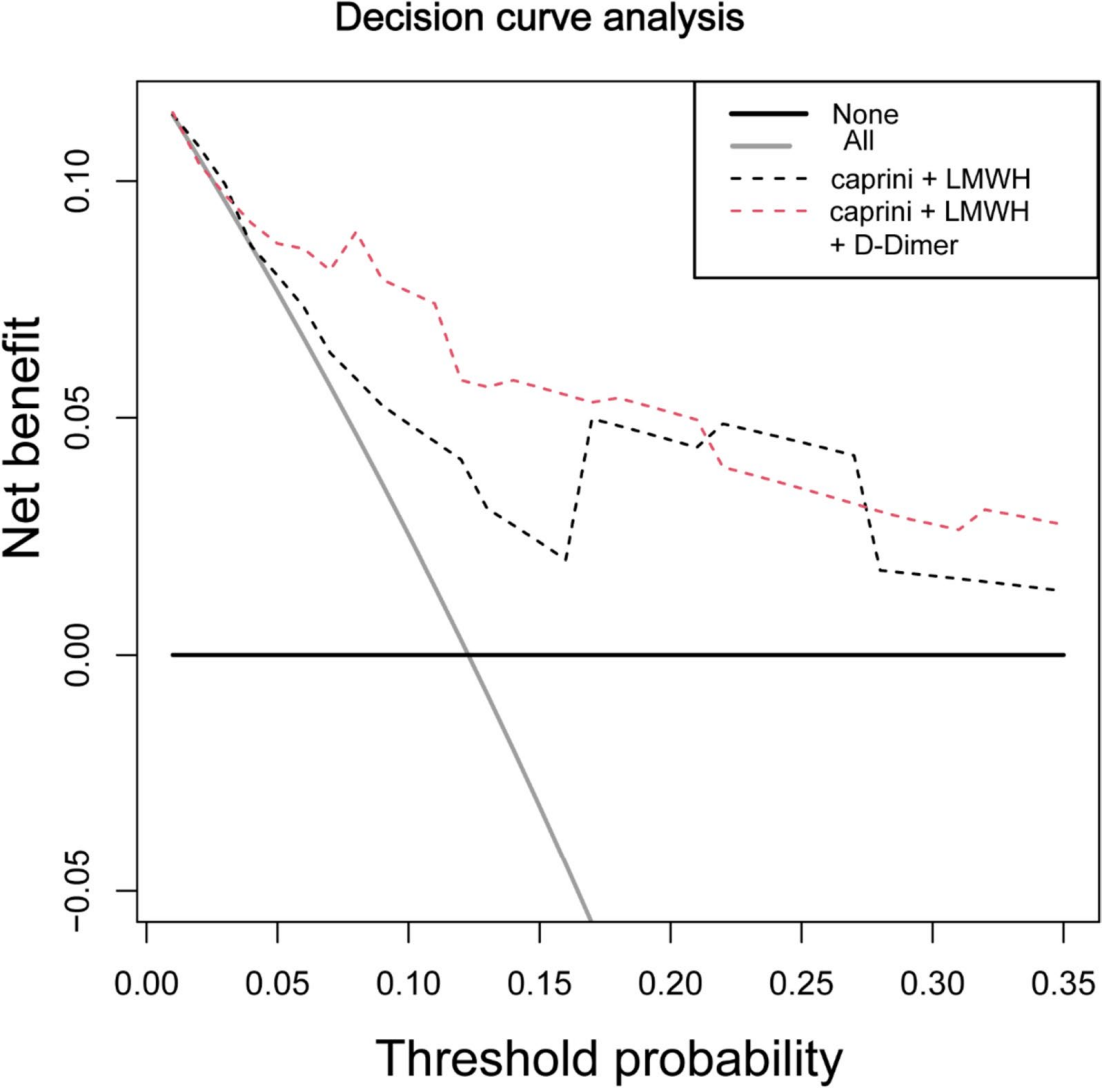
Caprini risk assessment model and LMWH can benefit patients more when combined with D-Dimer

The R language was used to make the decision curve model, with the threshold probability as Abscissa and benefit degree as ordinate [10]. Although the benefit degree of the Caprini score combined with the D-dimer level was between 21 and 27%, it was lower than that of the Caprini score alone; however, the overall trend was better than that of the Caprini score, particularly in the range of 16% of the first 5% of the curve, the benefit degree was much higher than that of the Caprini score alone. Concurrently, these two curves were above all the patients who received the intervention (Fig. 5).

**Fig. 6:**
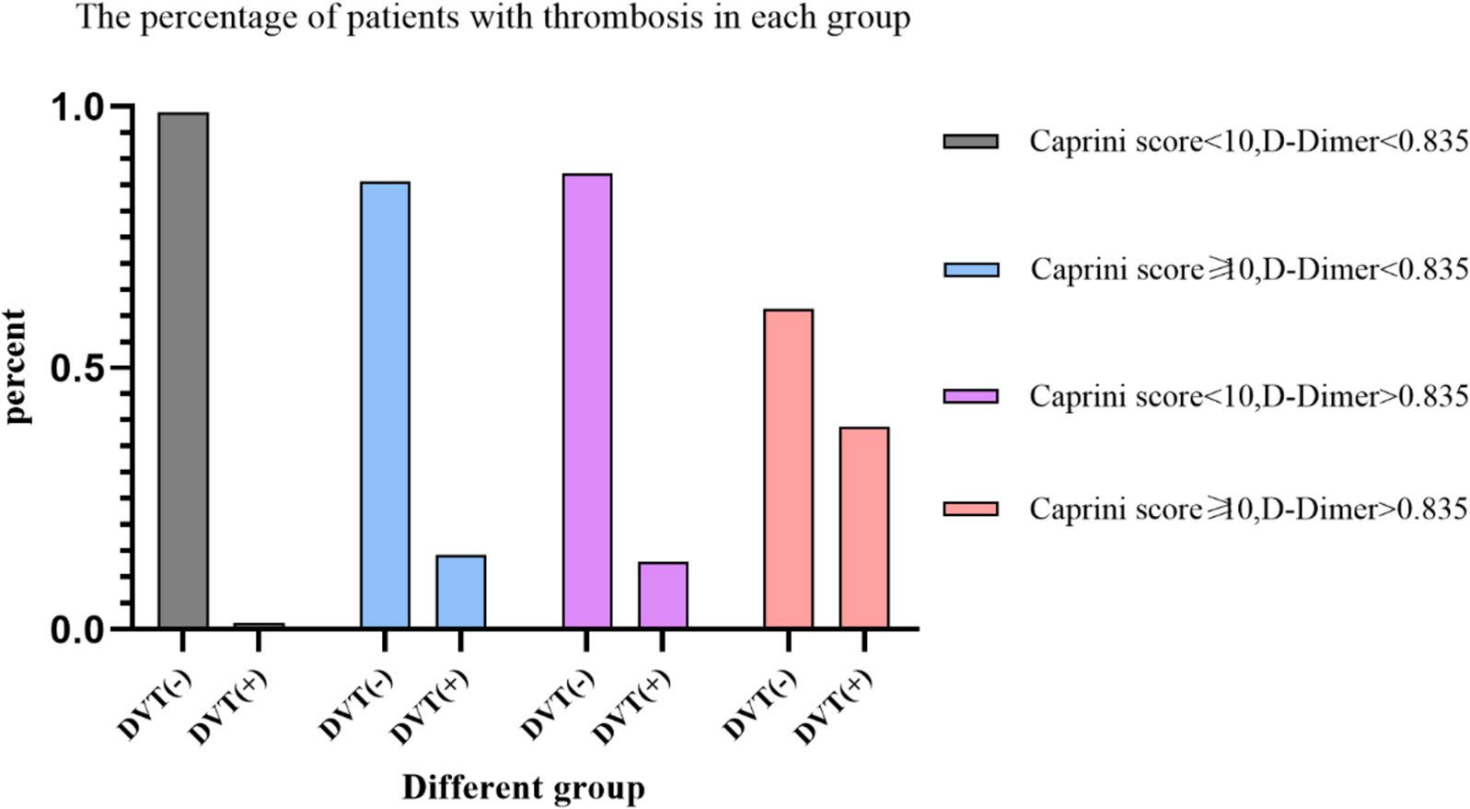
Bar plots of the patients with/without DVT in different groups stratified by Caprini score and D-dimer

Then, to facilitate potential clinical application, we divided the patients into four groups according to the boundary of a Caprini score of 9.5 and a D-dimer level of 0.835 μg/mL. The Caprini score could only be an integer; thus, we used a threshold of 10. The results revealed that patients with Caprini score < 10 and D-dimer < 0.835 have a very low probability (1.23%) of deep vein thrombosis. The incidence of DVT rose accompanied with increased Caprini score or elevated D-dimer. Patients with Caprini score > 10 and D-Dimer > 0.835 had the highest rate (38.7%) of DVT occurrence (Fig. 6). The incidence of DVT in the IPC + LMWH group was significantly lower than that in the IPC group for patients with Caprini score > 10 and D-dimer > 0.835 (16.7% vs 69.2%, *P* = 0.008).

## Discussion

Accurate recognition and prompt intervention are crucial for DVT prevention in perioperative patients with colorectal cancer. Our results confirmed that Caprini score and D-dimer were both independent risk factors for DVT. Combination of Caprini score and D-dimer had superior predictive ability than Caprini score or D-dimer alone. The incidence of thrombosis is very low in patients with a Caprini score of < 10 and a D-dimer level of < 0.835 μg/mL. Patients with higher Caprini scores and elevated D-dimer levels are most likely to develop DVT postoperatively. Anticoagulants should be strongly recommended during hospitalization for patients with a Caprini score ≥ 10 or a D-dimer > 0.835 μg/mL.

The applicability of the Caprini score in patients with colorectal cancer has been verified by predecessors. The AUC of lower extremity venous thrombosis predicted by the Caprini score in all patients with colorectal cancer was 0.701 [11]. We reached a similar conclusion that the AUC was 0.732 (95% CI 0.613–0.85) in all patients. In addition, we noted that the AUC was 0.792 (95% CI 0.639–0.945) in the IPC group and the Caprini score was moderately effective in patients with colorectal cancer. The positive predictive value of Caprini score in the IPC group was 52.2%, while adding D-dimer in the model improved the positive predictive value to 69.2%. Further studies are needed to further improve the applicability of Caprini risk assessment model.

The cutoff of Caprini score for patients with colorectal cancer might also need further discussions and investigations. In our study, all patients with colorectal cancer had a postoperative Caprini score of > 5, which meant that all patients needed postoperative anticoagulants. In fact, prophylactic anticoagulants have no significant effect on the occurrence of pulmonary embolism and proximal venous thrombosis of lower extremities (IPC vs. IPC + anticoagulant therapy = 5.1% vs. 2.76%, *P* = 0.382) [12]. Simultaneously, a previous meta-analysis of abdominal surgery found that it was important to use anticoagulants to prevent venous thrombosis in patients with a perioperative Caprini score of ≥ 7 score, whereas it was not required in patients with a Caprini score < 7, and it was recommended to further screen high-risk patients by increasing the threshold of the Caprini score [13]. It is possible to more accurately guide the use of postoperative anticoagulants in patients with colorectal cancer by adjusting the threshold of Caprini scores or by introducing new indicators.

D-dimer level tested by the enzyme-linked immunosorbent assays method has high sensitivity (95%) but low specificity (40%) when the cutoff value is 0.5 μg/mL [7]. In the IPC group, the positive and negative predictive value of D-dimer was 47.8% and 93.5%, correspondingly. It meant that elevated D-dimer level might not indicate incidence of DVT, while patients with D-dimer < 0.835 μg/mL could exclude possibility of DVT, At present, patients with colorectal cancer and lower postoperative D-dimer level should be considered low risk individuals for venous thrombosis [14]. In our study, we used D-dimer level on the first day after surgery as an indicator. Postoperative measurement of D-dimer before the use of anticoagulant drugs did not affect the course of anticoagulant therapy, but reflected the coagulation status of patients after surgery. There were 81 patients with Caprini score < 10 and D-dimer < 0.835 μg/mL, of which only one patient (1.23%) developed a DVT postoperatively, whereas the incidence of thrombus was higher in other groups. According to ACCP guideline, IPC is the best prophylaxis measure for patients with a low risk of venous thrombosis (< 1.5%) [6]. Preoperative use of anticoagulant drugs increased the risk of intraoperative and postoperative bleeding, but could not reduce the occurrence of postoperative thrombosis [15–17]. The application of LMWH could increase the incidence of clinical-related bleeding to 11.5% (60/524) [18]. Taking potential bleeding risk of LMWH into consideration, we do not recommend preventive prophylactic anticoagulants for these patients with colorectal cancer.

By introducing D-dimer level in the model, the Caprini score could more accurately predict the formation of DVT. Previously, the Caprini score combined with the D-dimer level showed higher predictive effectiveness in patients with thoracolumbar fractures caused by high-energy injuries [19]. We reached a similar conclusion that the AUC reached 0.865 after adding the D-dimer level to the Caprini ROC curve. When the Caprini score ≥ 10 and D-dimer > 0.835 μg/mL, the incidence of thrombosis was 38.7% (12/31), 16.7% in the IPC + LMWH group, and 69.2% in the IPC group (*P* < 0.01); thus, this group of patients needed the maximum amounts of prophylactic anticoagulants. Prolonged anticoagulants 4 weeks after laparoscopic colorectal cancer surgery can reduce the incidence of venous thrombosis from 9.7 to 0.9% [20]. We recommend that the drug prophylaxis be extended to 4 weeks for this group of patients.

Introducing D-dimer in thrombosis-related scoring system might be beneficial. The modified Geneva score and the Wells score are widely-used scoring system in pulmonary thrombosis [21]. Adding adjusted D-dimer level in the scoring system could improve the efficiency of the model [8, 21]. Adjusted D-dimer indicated high sensitivity for pulmonary thrombosis prediction, however, the specificity of D-dimer is low [8]. The unpromising specificity of D-dimer might limit its sole application in thrombosis prediction.

Of course, there are some limitations to our study. First, this is exploratory research. We were unable to preset the effect size and calculate the power of statistic in the study. Power analysis should be conducted in the future validation study. The effect size based on previous trails and the number of enrolled patients should be carefully designed. Second, the sample size of the single-center retrospective study was limited. Validation of the model based on Caprini and D-dimer in larger cohort should be done in the future. The study only performed internal verification; forward-looking experiments for external verification are still needed. The generalizability, reliability, and robustness of the model should be confirmed in diverse patient populations. Then, the potential false positive and false negative results of the model should be considered. False positive results might increase application of anti-coagulants drugs and potential bleeding risk. False negative results might increase risk of DVT for perioperative patients with CRC. Finally, the results of this study were only observed on the 6 (± 1) day postoperatively, excluding the occurrence of DVT after discharge. Actually, about 29% of DVT patients developed post-discharge thrombosis [22]. Delayed cases of DVT should not be neglected. Future studies of longer observation period should be done to provide a more comprehensive assessment of risk. Nonetheless, we improved the DVT predictive model based on Caprini score by introducing D-dimer, and provided more accurate stratification of DVT risk for patients with colorectal cancer. Different intervention methods should be considered for patients with different DVT risks.

## Conclusion

The Caprini risk assessment model and D-dimer can provide more accurate risk stratification for patients after laparoscopic radical resection of colorectal cancer.

## Data Availability

The original contributions presented in the study are included in the article/Supplementary Material, further inquiries can be directed to the corresponding author.

## Abbreviations

DVT: Deep vein thrombosis
IPC: Intermittent pneumatic compression
LMWH: Low molecular weight heparin
ACU: Area under curve
ROC: Receiver operating characteristic curve
